# Ultra-Precision Measurement and Control of Angle Motion in Piezo-Based Platforms Using Strain Gauge Sensors and a Robust Composite Controller

**DOI:** 10.3390/s130709070

**Published:** 2013-07-15

**Authors:** Lei Liu, Yu-Guang Bai, Da-Li Zhang, Zhi-Gang Wu

**Affiliations:** 1 State Key Laboratory of Structural Analysis for Industrial Equipment, Faculty of Vehicle Engineering and Mechanics, Dalian University of Technology, Dalian 116024, China; E-Mails: baiyg@dlut.edu.cn (Y.-G.B.); wuzhg@dlut.edu.cn (Z.-G.W.); 2 School of Aeronautics and Astronautics, Faculty of Vehicle Engineering and Mechanics, Dalian University of Technology, Dalian 116024, China; E-Mail: zdl@dlut.edu.cn

**Keywords:** piezo-based platform, ultra-precision angle motion, robust composite control

## Abstract

The measurement and control strategy of a piezo-based platform by using strain gauge sensors (SGS) and a robust composite controller is investigated in this paper. First, the experimental setup is constructed by using a piezo-based platform, SGS sensors, an AD5435 platform and two voltage amplifiers. Then, the measurement strategy to measure the tip/tilt angles accurately in the order of sub-μrad is presented. A comprehensive composite control strategy design to enhance the tracking accuracy with a novel driving principle is also proposed. Finally, an experiment is presented to validate the measurement and control strategy. The experimental results demonstrate that the proposed measurement and control strategy provides accurate angle motion with a root mean square (RMS) error of 0.21 μrad, which is approximately equal to the noise level.

## 1. Introduction

Ultra-precision angle motion and positioning have significant importance in free-space laser communication, space telescopes, staring cameras and some other space optical instruments [1–4 ]. For instance, the angle pointing accuracy of transfers and receivers should be kept in the order of micro-rad (μrad) to achieve free-space laser communication among satellites. Until recently, the problems of accurate angle motion and positioning in space applications were still not resolved well. To provide the angle motion on the order of μrad, a measurement and control strategy for a piezo-based two-degree-of-freedom (2-DOF) platform is investigated in this paper by employing SGS sensors which have been widely used for displacement measurement in the nanometer range. Intelligent actuators, which are commonly used to provide precision motion [5–7 ], are used to replace classical electric motors in this paper. Various intelligent actuators have been investigated for space applications [8–11 ], e.g., piezoelectric actuators, SMA and magnetostrictive actuators. Among these actuators, piezo actuators are increasingly being considered as core components due to their properties, including high accuracy, high speed and small size. Four piezoelectric actuators are used in this paper to achieve accurate angle motion. A novel driving principle of the piezo-based platform is employed. Only one DC voltage and two varying voltages are used to drive four piezo stacks.

Additionally, high bandwidth angle measurement on the order of sub-μrad is necessary in free-space optics applications. For example, angle acquisition and tracking with a bandwidth of hundreds of Hz is required in the acquisition, tracking and pointing (ATP) systems of space laser communications. Generally, the tip and tilt (also named pitch and yaw) angles are measured and determined by attitude sensors, e.g., sun sensors, star sensors, integrating gyros and fiber optic gyros, but the accuracy is generally worse than 10 μrad and the measurement bandwidth is generally less than 5 Hz. To measure tip and tilt angles on the order of sub-μrad, SGS sensors are employed in this paper to determine the angles with high accuracy and high bandwidth by measuring the length changes of piezo actuators. Then, the tip and tilt angles are computed according to these length changes. Meanwhile, electric bridges can be chosen for better stability and resolution [12 ].

To achieve fast angle motion in the order of sub-μrad, closed loop control is also necessary because of the linear dynamics and hysteresis effects in the piezoelectric actuators [13–15 ]. It is known that the open loop errors of piezoelectric actuators can be as large as 10%–15% of their travel span, even at low frequencies (*i.e.*, less than 0.1 Hz). Some compensation approaches for the hysteretic dynamics of piezo systems have been proposed [16–19 ]. In this paper, a comprehensive composite control strategy consisting of the proposed robust *H*_∞_ feedback controller and derivative feedforward controller is designed to efficiently compensate the linear dynamics and hysteresis effects. Instead of complex hysteretic dynamics, linear dynamics is used to represent the piezo-based platform. The composite controller is designed from an application perspective. Compared with most other *H*_∞_ works, the trade-offs between the control bandwidth, measurement noise and control limitation have been quantitatively considered in our work. Moreover, a realtime physical simulation platform (*i.e.*, AD5435) is employed to provide realtime closed loop control. The tracking error of the proposed robust composite control method approaches the measurement noise level.

This paper is organized as follows: firstly, the experimental setup and working principle are presented in Section 2. Next, the measurement scheme of the tip/tilt platform is proposed in Section 3. A piezo-based platform, an AD5435 realtime platform, two amplifiers and SGS sensors are employed. Then, the composite control system of the piezo-based platform is presented in Section 4. Finally, the experimental results and discussions are presented.

## 2. System Description

### 2.1. Experimental Setup

**Figure 1.**
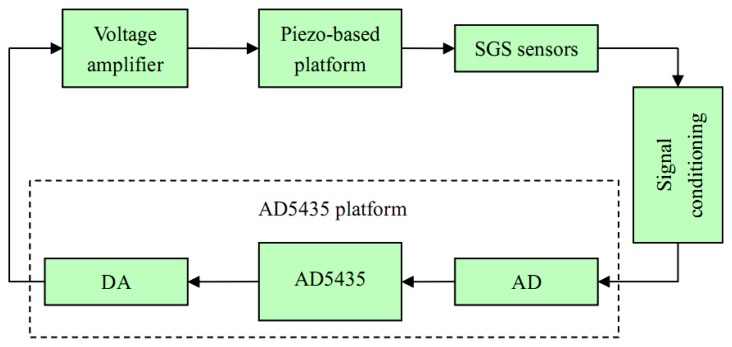
Experimental components for precision angle motion.

The experimental setup, the measurement strategy and the angle calibration are presented in this section. The constructed piezo platform system consists of a piezo-based platform, SGS sensors, voltage amplifiers, an AD5435 platform and a signal conditioning unit, as shown in Figure 1. The AD5435 platform includes an AD5435 card, a DA card (AD5430-02BK, 8 Ch, 16 Bits) and an AD card AD5430-01, 16 Ch, 16 Bits). In addition, voltage amplifiers, SGS sensors and signal conditioning are installed in a metallic box. The bandwidth of SGS sensors in this paper can be up to 3 kHz, and the resolution is better than 10 nm (RMS). The equivalent angle resolution is 0.2 μrad (RMS).

**Figure 2.**
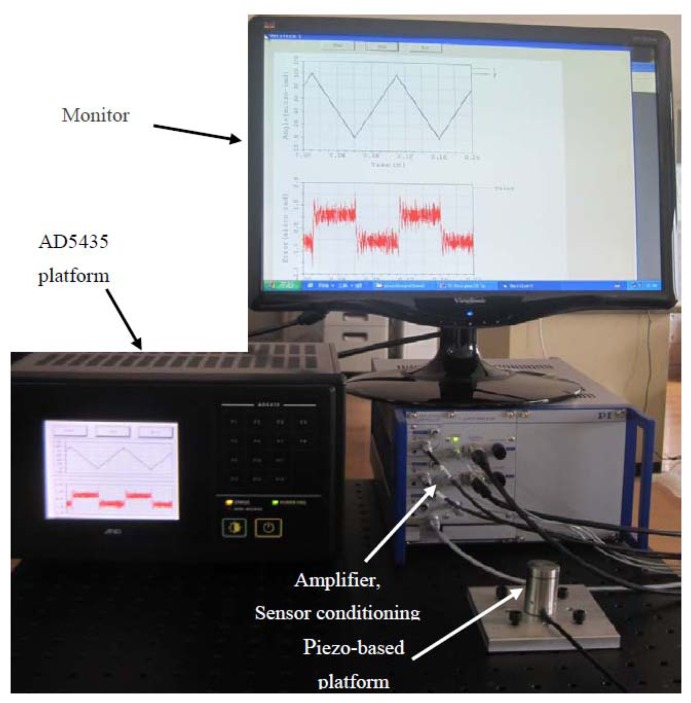
Experimental setup.

The experimental setup is shown in Figure 2. The piezo-based platform system for experiments is constructed to achieve precision angle motion on the order of sub-μrad.

### 2.2. Working Principle

**Figure 3.**
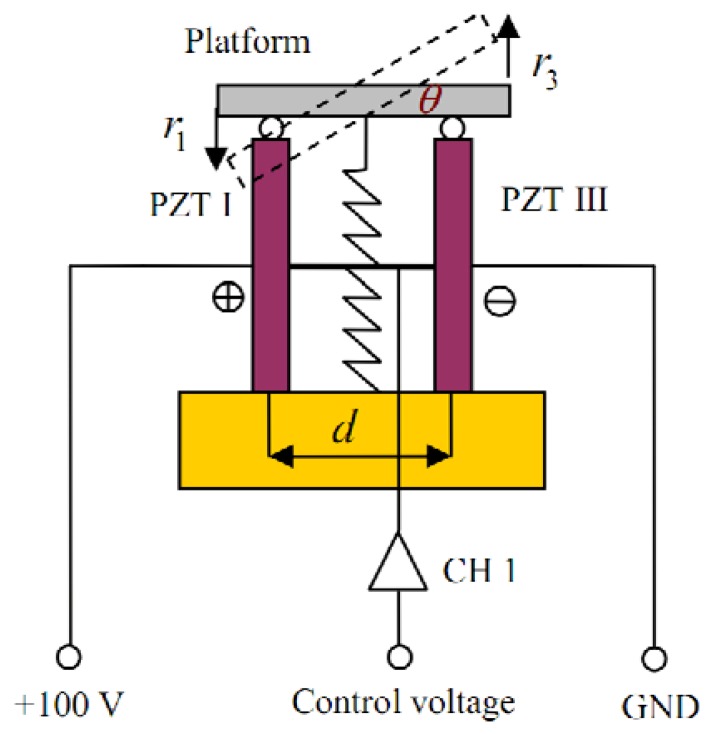
Working principle of the piezo-based platform in X-axis.

The working principle of the proposed piezo-based platform is different from that of other piezo applications. First, a preloading spring is used to maintain the structure stability. Then, a constant voltage and two varying inputs are used to drive four independent piezo stacks. The working principle of the piezo-based platform in X-axis is shown in Figure 3, where *θ*, *r*_1_ and *r*_3_ are the tilt angle, the translational displacements of PZT I and PZT III, respectively. A constant voltage +100 V is added to PZT I. Thus, the input voltage of CH 1 can be used to drive both PZT I and PZT III to produce the tilt angle *θ*.

**Figure 4.**
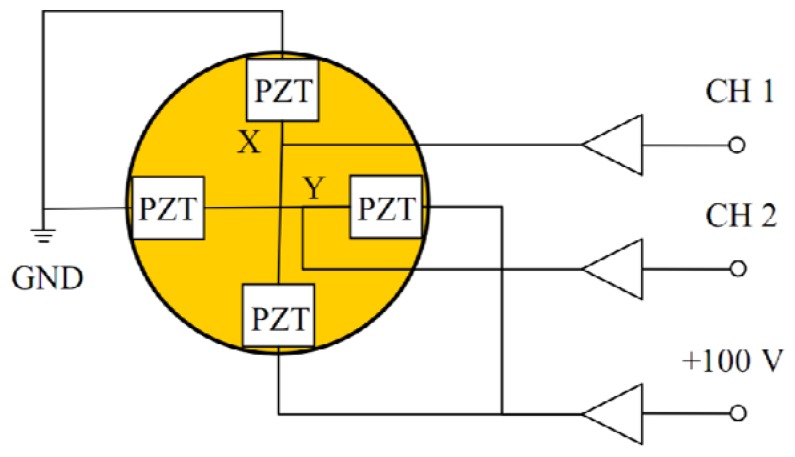
Working principle of the piezo-based platform in both X-axis and Y-axis.

To further introduce the working principle, Figure 4 shows the working principle of the piezo-based platform in both X-axis and Y-axis. CH 1 and CH 2 can be used to drive the four PZT stacks because the constant voltage +100 V are preloaded into two of the PZT stacks.

## 3. Measurement Strategy

In this section, the measurement strategy to provide angle measurements with high accuracy on the order of sub-μrad is presented. Instead of direct measurements of the angle motion, the positions of PZT stacks are measured first. Then the angle motion is computed. To evaluate the accuracy of the measurement strategy, the angle calibration is also presented.

### 3.1. Measuring Principle of SGS Sensors

**Figure 5.**
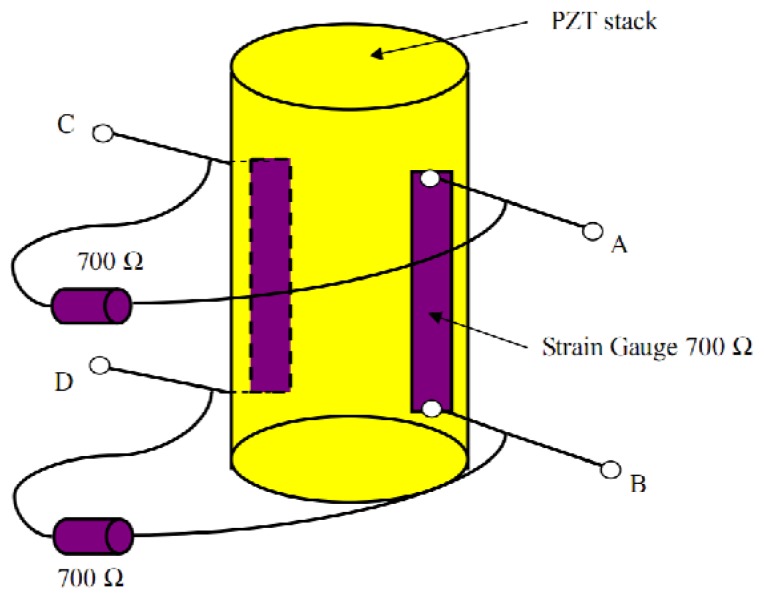
Measuring principle of the PZT stack position using SGS sensors.

SGS sensors are widely applied to measure piezo displacements. In this paper, one piezo actuator (PZT stack) is used to show the measuring principle of SGS sensors. Two resistive films are bonded to the PZT stack, as shown in Figure 5. The length change of the piezo stack alters the resistive of the strain gauge. The resistive change is proportional to the displacement of the PZT stack. Moreover, a Wheatstone electric bridge, which consists of two strain gauges on the PZT stacks and two resistors on the PZT housing, is employed to get better stability and resolution.

The strain gauges are used to measure the mechanical length change, because the length change results in the resistance change and the generation of voltage signal. More details of the electric bridges are presented by Huang *et al.* [12 ].

**Figure 6.**
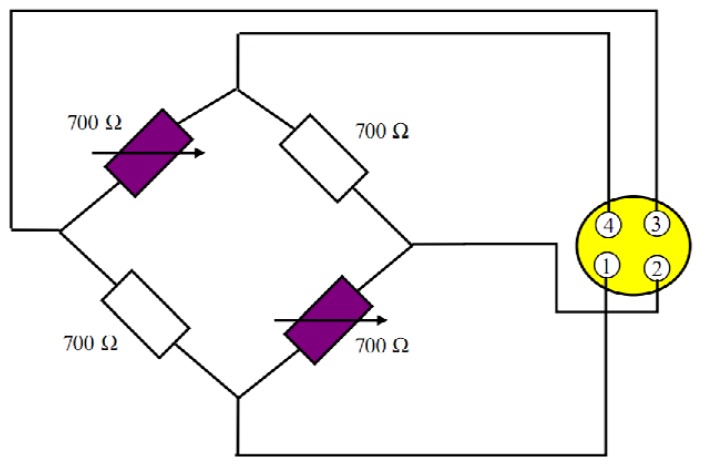
Measuring principle of the PZT stack position using SGS sensors.

Figure 6 shows the sketch of the resistance bridge. Four resistors are used. Two of them are active and bonded to the PZT stack and the other two are bonded to the housing. When the piezo length changes, a stress is applied to the bonded strain gauge. Then, the Wheatstone bridge becomes unbalanced and a resistive change takes place, so a voltage signal is generated and it is proportional to the length change. With the conditioning electronics, the voltage is amplified to 0–10 V.

### 3.2. Measuring Principle of Tip/tilt Angles

The tip/tilt angles of the piezo-based platform are indirectly measured by comparing the length changes of the PZT stacks. First, the SGS sensor gives the length change of the PZT stack. The relationship among *θ*, *r*_1_ and *r*_3_ in Figure 3 can be represented as:

(1) $$\tan\theta =\frac{r_{1}+r_{3}}{d}$$

The travel span of the piezo stacks in this paper is 15 μm. The max tip/tilt angles are 0.002 rad. Thus, tan *θ* ≈ *θ*. So *θ* can be represented by the following Equation (2);

(2) $$\theta =\frac{r_{1}+r_{3}}{d}$$

Finally, there is a linear relationship between the tilt angle and the piezo displacements and it will be validated in the next calibration section.

### 3.3. Angle Calibration

To validate the measurement strategy the angle calibration is presented. For simplification, only the tilt angle calibration is presented. A ZYGO ZMI-2000 interferometer is used as the measurement setup to calibrate angles. The calibration temperature is 20.8 °C and the calibration humidity is equal to 39%.

**Figure 7.**
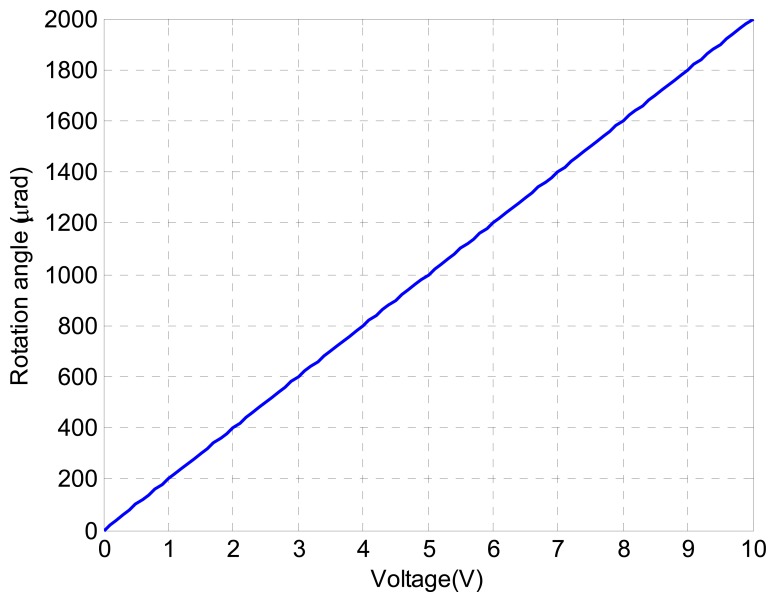
Relationship between the sensor output voltage and its angle displacement.

Figure 7 shows the relationship between the sensor output voltage and the angle displacement. It can be seen that there exist quasi-linear relationship between the sensor output voltage and the angle displacement.

**Figure 8.**
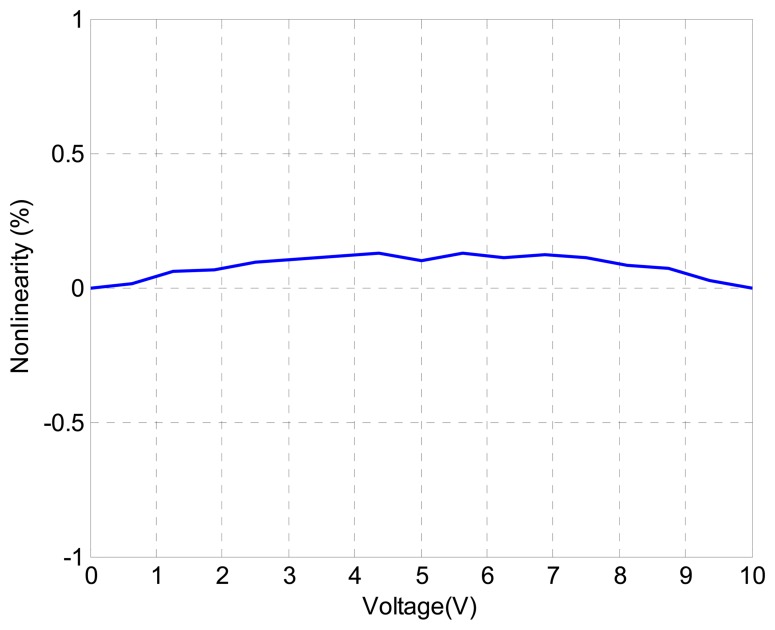
Nonlinearity of the measurement of SGS sensors.

Furthermore, Figure 8 shows the nonlinearity of the angle displacement measurement with respect to the sensor output voltage. The nonlinearity is represented by the relative error (%). It can be seen that the nonlinearity of the measurement is less than 0.1%. This demonstrates that the measurement strategy in this paper to measure the angle motion of the piezo-based platform is accurate and effective.

## 4. Control System Design

The comprehensive design of the composite control of the piezo-based platform to achieve precision angle motion is proposed in this section. In this section, only the tilt motion (*i.e.*, in X-axis) control is investigated. The coupling effect of tilt and tip motion of the piezo-based platform is represented by an input uncertainty. Firstly, the linear dynamics of the piezo-based platform are identified. Then, the robust *H*_∞_ feedback controller is designed. Sequentially, the derivative feedforward is incorporated to enhance the tracking performance. Finally, the proposed composite controller is applied to the piezo-based platform to demonstrate the tracking performance.

### 4.1. Modeling and Identification

Modeling and identification are necessary for the design of a modern robust control system. Various methods for modeling and identifiying piezo systems have been investigated [20–23 ]. In order to design a robust optimal *H*_∞_ controller, time-invariant linear dynamics are used to represent the main dynamics of the PZT stacks. Also, the un-modeled dynamics is represented by an input uncertainty.

The time-invariant linear dynamics of the piezo-based platform is identified by using the square wave input (only X-axis is investigated). By employing armax method (*i.e.*, auto regressive moving average models with external input), the linear dynamics of the piezo stack is identified and represented by the following transfer function:

(3) $$G=422\frac{\left(s^{2}+306.7s+5.022\times 10^{6})(s^{2}-5.605\times 10^{4}s+7.938\times 10^{8})(s^{2}+2358s+1.052\times 10^{8}\right)}{\left(s+1996\right)(s^{2}+1021s+5.171\times 10^{6})(s^{2}+5816s+6.632\times 10^{7})(s^{2}+6364s+3.156\times 10^{8})}$$

where *G* represents the time-invariant linear dynamics of the piezo-based platform system and *s* denotes a differentiation operator.

### 4.2. Design of Robust H_∞_ Optimal Control

Multi-objective robust *H*_∞_ optimal control is investigated in this section in order to achieve precision angle motion in the face of modeling uncertainty and measurement noise. Weighting functions and loop shaping technique are used to design the robust *H*_∞_ controller.

**Figure 9.**
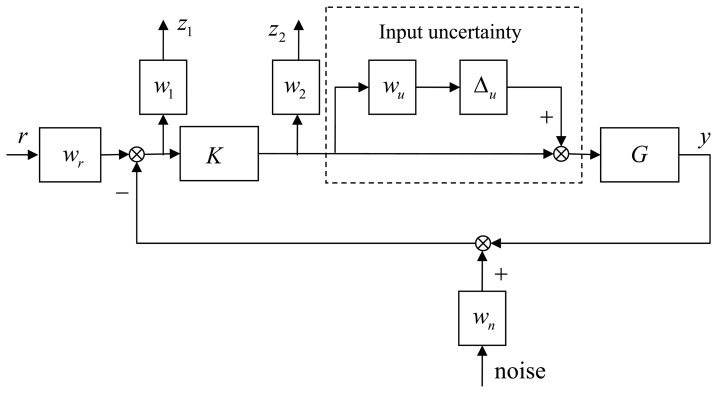
Design sketch of the robust *H*_∞_ controller.

Figure 9 shows the design sketch of the robust *H*_∞_ control system, in which *K* and *G* represent the robust *H*_∞_ controller and the linear dynamics of the PZT stacks, respectively, and *w*_1_ is the performance weighting function. To enhance the disturbance suppressing at low frequencies and limit the feedback gain at high frequencies simultaneously, an integral action is added to *w*_1_, and *w*_2_ is the control weighting function which limits the control gain at high frequencies. To guarantee the robust stability at high frequencies, the control signal is limited to slower than 2,000 Hz. The terms *w_r_* and *w_n_* represent the reference and noise weighting functions, respectively. In the experiments, the measurement noise level at frequencies higher than 500 Hz is 100 times the noise level at frequencies lower than 5 Hz. The weighting function *w_u_* denotes the modeling uncertainty mainly produced by the hysteresis nonlinearity of the PZT stacks; and ∆*u* is an unit complex dynamic uncertainty with ║*∆u*║ < 1 After measuring the hysteretic loop in experiments, the weighting function *w_u_* is set to 5%. After several trials, the weighting functions *w_r_*, *w_u_*, *w*_1_, *w_2_* and *w_n_* are designed and represented in the following Equations (4-7):

(4) $$w_{r}=1,w_{u}=0.05$$

(5) $$w_{1}=\frac{3000}{s+0.0001}$$

(6) $$w_{2}=0.5{\left(\frac{s+400\pi }{s+400\pi }\right)}^{2}$$

(7) $$w_{n}=0.01\frac{s+10\pi }{s+1000\pi }$$

The *D-K* iteration approach with structured singular values is used to solve the *H*_∞_ optimal controller [24 ]. After 10 iteration steps, the final structure singular value *μ* is equal to 0.95 which is less than 1. The small gain theorem is satisfied and the *H_∞_* feedback control is stable. Moreover, its order is reduced to 8, so it is easier to implement the *H_∞_* controller in an AD5435 card. The reduced *H_∞_* optimal controller is represented in the following Equation (8):

(8) $$K=2.04\frac{\left(s+2215)(s^{2}+1119\;s+4.872\times 10^{6})(s^{2}+4579\;s+8.388\times 10^{7})(s^{2}+6893\;s+4.233\times 10^{9}\right)}{\left(s+0.0001\right)(s^{2}+375.9\;s+4.943\times 10^{6})(s^{2}+3079\;s+9.535\times 10^{7})(s^{2}+6359\;s+3.958\times 10^{9})}$$

### 4.3. Composite Control Design

**Figure 10.**
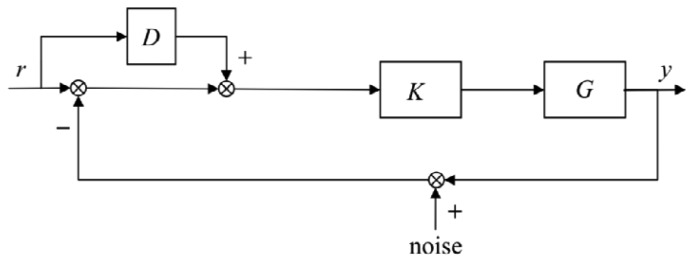
Composite control sketch of the piezo-based platform.

To further enhance the tracking performance of the robust *H*_∞_ feedback control in Section 4.2, a derivative feedforward of the reference signal is added to the tacking error. The composite control is thus designed, as shown in Figure 10, where *D* and *K* represent the derivative feedforward controller and the robust *H*_∞_ feedback controller, respectively, and *r* and *y* represent the reference angle and piezo angle, respectively. In the experiment, *D* is determined by several trials.

Finally, *D* is represented as follows:

(9) D=0.0004s

where s represent the derivative operator.

**Figure 11.**
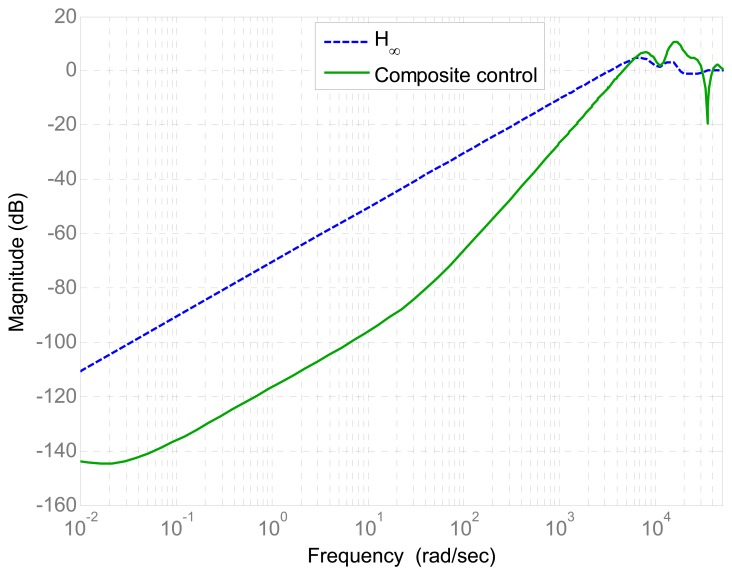
Bode diagrams of the sensitivity function of the *H*_∞_ controller and composite controller.

In this paper, the derivative feedforward *D* is employed to compensate the phase lag within the feedback bandwidth. By transformation, the proposed composite controller can be regarded as a special PD-*H*_∞_ composite controller. To represent the tracking performance in frequency domain, the Bode diagram of the sensitivity functions of the *H*_∞_ controller and composite controller is shown in Figure 11. It can be seen that the proposed composite controller greatly improve the tracking performance at frequencies lower than 3,500 rad/s. At frequencies higher than 5,000 rad/s, the tracking performance of the proposed composite controller does not hold. Alternatively, the robust stability is guaranteed by the robust *H*_∞_ feedback controller at frequencies higher than 5,000 rad/s.

### 4.4. Simulation Result of the Proposed Composite Controller

**Figure 12.**
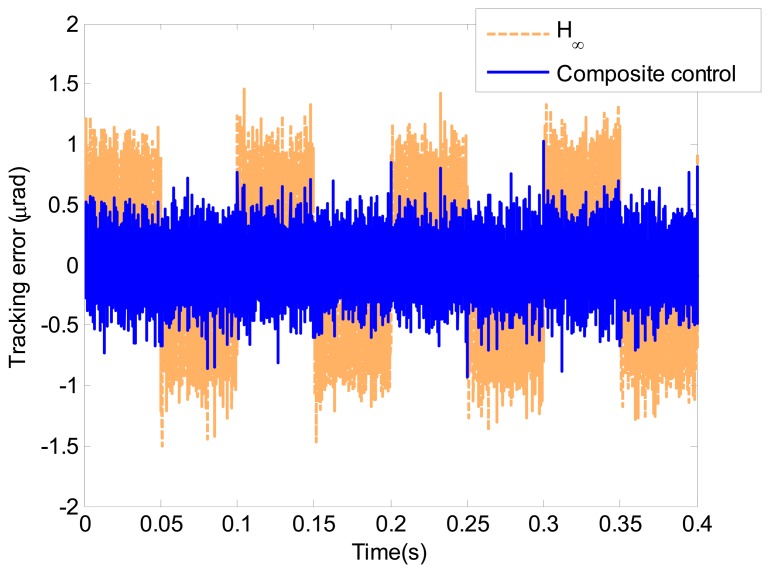
Tracking errors in simulation study.

In this section, the simulation study of a triangle wave with the amplitude of 100 μrad and the frequency of 10 Hz is presented. Figure 12 shows the tracking errors of the proposed composite controller and the *H*_∞_ controller. The tracking error of the proposed composite control is less than 36% of the *H*_∞_ feedback control. The tracking error RMS of the proposed composite control is 0.205 μrad, which is almost equal to the electrical noise. The simulation result indicates that the proposed composite controller presents satisfactory tracking performance.

## 5. Experimental Results and Discussion

### 5.1. Experimental Results

**Figure 13.**
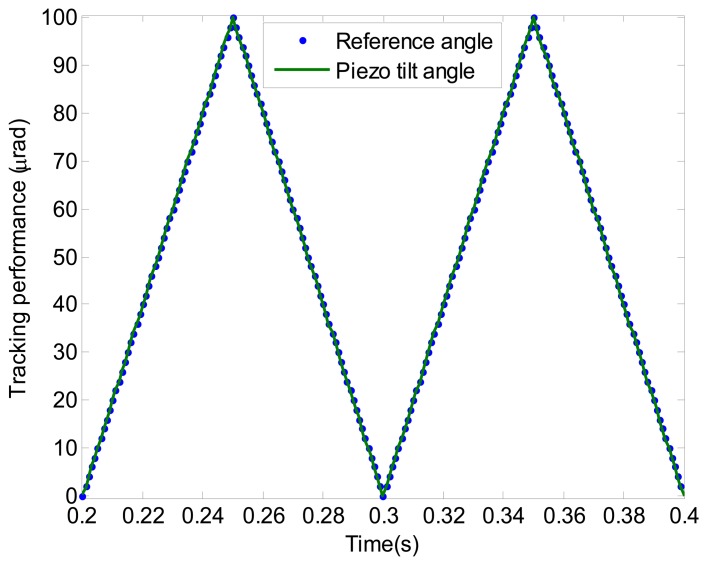
Tracking performance of the triangle wave.

In this section, the experimental studies to demonstrate the measurement and control strategy are described. Both triangle and sinusoidal waves are selected as the reference signal. Firstly, the triangle wave at 10 Hz is chosen as the reference signal *r*. Figure 13 shows the tracking performance of the proposed composite control. It can be seen that the piezo-based platform tracks the reference angle with satisfactory performance.

**Figure 14.**
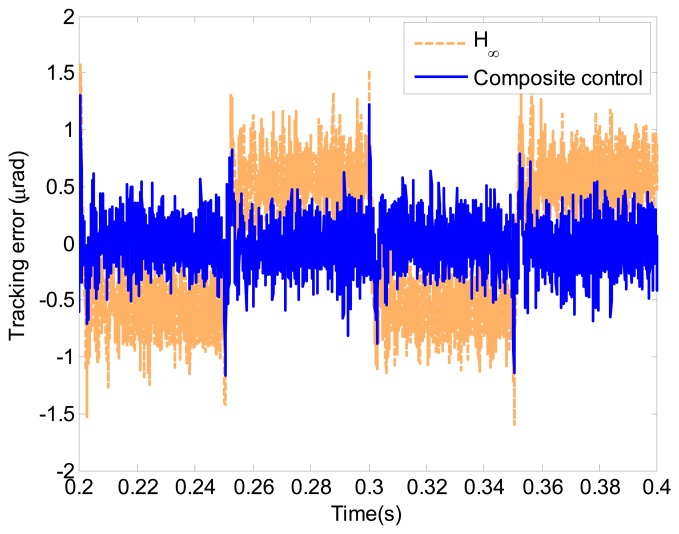
Tracking error of the triangle wave in the piezo experiments.

The tracking error of the proposed composite control is shown in Figure 14. Additionally, the tracking error is given in Figure 14. Compared with the simulation result in Figure 12, the performance degradation of the *H*_∞_ and composite control is negligible. The tracking error of the proposed composite control is less than 0.8 μrad with the RMS error is 0.21 μrad. The relative tracking error of the proposed composite control is 0.36% of the reference signal RMS. The following Equation (9) is used to represent the relative tracking error.

**Figure 15.**
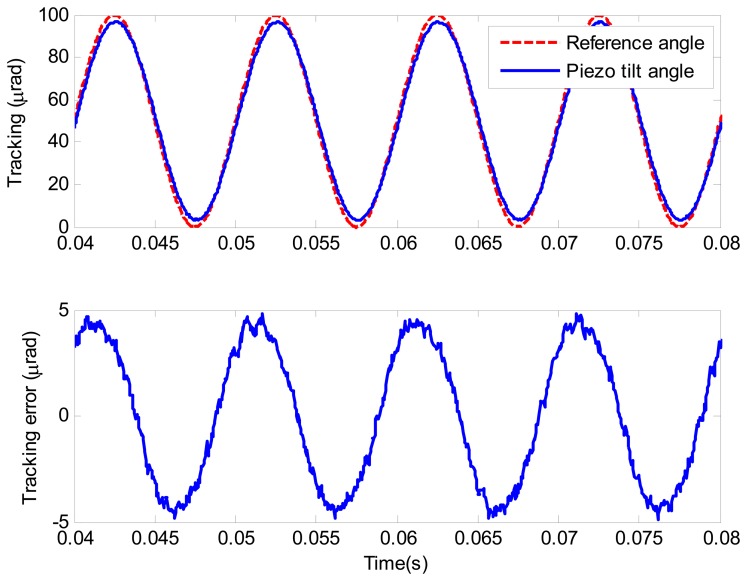
Tracking performance of sinusoidal wave at 100 Hz under the composite control.

The following Equation (9) is used to represent the relative tracking error:

(9) $$e_{\text{rms}}=\left(\frac{\sqrt{\frac{1}{n}\sum_{i=1}^{n}(y_{r}(i)-y(i))}}{\sqrt{\frac{1}{n}\sum_{i=1}^{n}y_{r}(i)}}\right)\times 100\%$$

where *n* is the sampling number, *y_r_* is the reference angle, and *y* is the output angle. To further demonstrate the high frequency tracking performance of the proposed composite control, the tracking of a sinusoidal wave at 100 Hz is given in Figure 15. It can be seen that satisfactory performance is presented. The relative tracking error of the proposed composite control is 5.7%.

### 5.2. Discussion

The experimental results reveal that the precision angle motion of the piezo-based platform can be achieved by employing the proposed composite controller. In addition, for angle motion and positioning with high accuracy, linearity and repeatability in the order of sub-μrad, closed loop operation is necessary for the piezo-based platform, though the piezo-based platform is commonly regarded as a precision system. Thus a robust composite controller is designed in closed loop to compensate the linear dynamics and hysteresis effects. It can be seen from the results that the tracking accuracy of the triangle wave at 10 Hz approaches the level of measurement noise.

As for comparison, the experimental results in this paper are compared with that in Reference [18 ], where the complex hysteresis effect is modeled and compensated. In Reference [18 ], the relative tracking error at 0.01 Hz is 4.37% by using a composite controller consisting of an inverse-Preisach hysteresis and PD/lead-lag feedback. In this paper, the relative tracking error at 10 Hz with the proposed composite control is as small as 0.37%. Furthermore, the reference signal is faster than that in Reference [18 ], where the maximum testing frequency is 0.1 Hz, but the maximum reference frequency in this paper is 100 Hz. It indicates the proposed composite control in this paper provides more accurate tracking at higher bandwidth.

## 6. Conclusions

Precision angle motion and positioning in space optics are increasingly required. A piezo-based platform system is constructed in addition to a measurement and control strategy to resolve this problem. A measurement strategy providing effective measurement with high bandwidth and accuracy is investigated. A comprehensive design of the composite control strategy is developed. The effectiveness of precision angle motion under the proposed composite controller is validated through the experimental results. It can be demonstrated from the results that the measurement and control strategy in this paper will have significant benefit for future applications in space optics.
